# Supplementary material for: Polymorphisms in ACE1, TMPRSS2, IFIH1, IFNAR2, and TYK2 Genes Are Associated with Worse Clinical Outcomes in COVID-19
Source: Genes (Basel). 2022 Dec 22;14(1):29. doi: 10.3390/genes14010029 (PMC9858252; doi:10.3390/genes14010029)
Supplement: Supplementary file 1 [file genes-14-00029-s001.zip › genes-2029564-supplementary.pdf]

Supplementary Table S1. Logistic regression data of the 9 analyzed polymorphisms in patients with COVID-19 according to disease severity and mortality.

Polymorphism	Inpatients *vs.* ICU patients	Survivors *vs.* non-survivors
	**Adjusted OR (95% IC) / P†**	**Adjusted OR (95% IC) / P†**
rs1799752 - *ACE1*		
Genotype	2.579 (0.823 – 8.083)/ 0.104	2.387 (0.814 – 7.617)/ 0.142
Recessive model	2.380 (0.779 – 7.272)/ 0.128	2.143 (0.690 – 6.651)/ 0.187
Additive model	2.586 (0.797 – 8.390)/ 0.114	2.307 (0.695 – 7.653)/ 0.172
Dominant model	1.157 (0.819 – 1.635)/ 0.407	1.196 (0.836 – 1.712)/ 0.327
rs2285666 - *ACE2*		
Genotype	1.173 (0.731 – 1.882)/ 0.509	1.246 (0.750 – 2.072)/ 0.396
Recessive model	1.208 (0.754 – 1.935)/ 0.431	1.285 (0.775 – 2.132)/ 0.331
Additive model	1.171 (0.728 – 1.881)/ 0.515	1.235 (0.741 – 2.055)/ 0.418
Dominant model	0.925 (0.656 – 1.304)/ 0.656	0.930 (0.651 – 1.329)/ 0.690
rs2109069 – *DPP9*		
Genotype	1.306 (0.728 – 2.344)/ 0.370	1.303 (0.694 – 2.448)/ 0.410
Recessive model	1.351 (0.769 – 2.374)/ 0.295	1.393 (0.757 – 2.562)/ 0.286
Additive model	1.289 (0.717 – 2.320)/ 0.397	1.293 (0.687 – 2.434)/ 0.426
Dominant model	0.987 (0.719 – 1.354)/ 0.935	0.925 (0.668 – 1.280)/ 0.638
rs1990760 – *IFIH1*		
Genotype	1.184 (0.746 – 1.879)/ 0.473	1.162 (0.723 – 1.868)/ 0.535
Recessive model	1.165 (0.780 – 1.740)/ 0.455	1.168 (0.771 – 1.768)/ 0.464
Additive model	1.258 (0.786 – 2.013)/ 0.339	1.227 (0.757 – 1.987)/ 0.407
Dominant model	1.070 (0.755 – 1.517)/ 0.702	1.039 (0.728 – 1.482)/ 0.834
rs223675 – *IFNAR2*		
Genotype	1.079 (0.648 – 1.796)/ 0.769	1.153 (0.684 – 1.943)/ 0.593
Recessive model	1.027 (0.635 – 1.661)/ 0.913	1.080 (0.659 – 1.768)/ 0.761
Additive model	1.079 (0.648 – 1.798)/ 0.770	1.150 (0.682 – 1.939)/ 0.601
Dominant model	1.097 (0.799 – 1.506)/ 0.565	1.144 (0.827 – 1.584)/ 0.417
rs368234815 – *IFNL4*		

Genotype	1.246 (0.793 – 1.956)/ 0.340	1.224 (0.769 – 1.949)/ 0.394
Recessive model	1.191 (0.788 – 1.801)/ 0.406	1.172 (0.766 – 1.791)/ 0.464
Additive model	1.273 (0.806 – 2.011)/ 0.300	1.254 (0.783 – 1.055)/ 0.681
Dominant model	1.134 (0.819 – 1.568)/ 0.449	1.126 (0.806 – 1.573)/ 0.487
rs3775291 – *TLR3*		
Genotype	0.908 (0.528 – 1.560)/ 0.726	1.003 (0.571 – 1.760)/ 0.992
Recessive model	0.843 (0.502 – 1.415)/ 0.517	0.943 (0.550 – 1.618)/ 0.832
Additive model	0.933 (0.541 – 1.609)/ 0.803	1.014 (0.575 – 1.787)/ 0.962
Dominant model	1.114 (0.812 – 1.527)/ 0.503	1.111 (0.804 – 1.537)/ 0.524
rs12329760 – *TMPRSS2*		
Genotype	0.841 (0.352 – 2.007)/ 0.696	0.865 (0.362 – 2.065)/ 0.744
Recessive model	0.786 (0.332 – 1.864)/ 0.585	0.807 (0.340 – 1.912)/ 0.625
Additive model	0.838 (0.349 – 2.016)/ 0.694	0.860 (0.358 – 2.065)/ 0.736
Dominant model	1.192 (0.856 – 1.660)/ 0.299	1.201 (0.854 – 1.688)/ 0.292
rs2304256 – *TYK2*		
Genotype	1.650 (0.764 – 3.565)/ 0.202	1.710 (0.759 – 3.851)/ 0.196
Recessive model	1.626 (0.761 – 3.474)/ 0.209	1.663 (0.747 – 3.705)/ 0.213
Additive model	1.661 (0.769 – 3.591)/ 0.197	1.702 (0.755 – 3.836)/ 0.200
Dominant model	1.093 (0.796 – 1.501)/ 0.583	1.127 (0.813 -* 1.561)/ 0.474

† P-values and OR (95% CI) obtained using logistic regression analyses adjusting for age, ethnicity and sex.

Supplementary Table S2. Genotype and allele frequencies of the analyzed polymorphisms in females with COVID-19 according to disease severity and mortality.

	Inpatients	ICU patients	Unadjusted P*	Adjusted OR (95% IC) /† P
**rs2285666 – *ACE2***	151	175		
**Genotype**				
C/C	79 (52.3)	105 (60.0)		1
C/T	64 (42.4)	58 (33.1)		0.730 (0.456 – 1.169)/0.190
T/T	5 (5.3)	12 (6.9)	0.221	1.190 (0.455 – 3.184)/0.728
**Allele**				
C	0.75	0.77		
T	0.25	0.23	0.709	
**Recessive model**				
C/C + C/T	143 (94.7)	163 (93.1)		1
T/T	8 (5.3)	12 (6.9)	0.724	1.348 (0.513 – 3.541)/ 0.544
**Additive model**				

C/C	79 (90.8)	105 (89.7)		1
T/T	8 (9.2)	12 (10.3)	0.989	1.221 (0.455 – 3.279)/ 0.692
**Dominant model**				
C/C	79 (52.3)	105 (60.0)		1
C/T + T/T	72 (47.7)	70 (40.0)	0.200	0.780 (0.497 – 1.225)/ 0.281
**rs2109069 – *DPP9***	151	175		
**Genotype**				
G/G	79 (52.3)	81 (46.3)		1
G/A	61 (40.4)	80 (45.7)		1.098 (0.686 – 1.756)/ 0.698
A/A	11 (7.3)	14 (8.0)	0.553	1.076 (0.456 – 2.540)/ 0.867
**Allele**				
G	0.73	0.69		
A	0.27	0.31	0.391	
**Recessive model**				

G/G + G/A	140 (92.7)	161 (92.0)		1
A/A	11 (7.3)	14 (8.0)	0.973	1.028 (0.449 – 2.355)/ 0.947
**Additive model**				
G/G	79 (87.8)	81 (85.3)		1
A/A	11 (12.2)	14 (14.7)	0.776	1.048 (0.442 – 2.486)/ 0.916
**Dominant model**				
G/G	79 (52.3)	81 (46.3)		1
G/A + A/A	72 (47.7)	94 (53.7)	0.329	1.094 (0.697 – 1.718)/ 0.695
**rs223675 – *IFNAR2***	151	175		
**Genotype**				
**G/G**	61 (40.4)	68 (38.9)		1
G/A	67 (44.4)	82 (46.9)		1.110 (0.685 – 1.798)/ 0.672
A/A	23 (15.2)	25 (14.2)	0.901	1.113 (0.556 – 2.228)/ 0.762
**Allele**				
**G**	0.63	0.62		

A	0.37	0.38	0.997	
**Recessive model**				
G/G + G/A	128 (84.8)	150 (85.7)		1
A/A	23 (15.2)	25 (14.3)	0.933	1.054 (0.553 – 2.009)/ 0.874
**Additive model**				
**G/G**	61 (72.6)	68 (73.1)		1
A/A	23 (27.4)	25 (26.9)	1.000	1.137 (0.568 – 2.277)/ 0.716
**Dominant model**				
**G/G**	61 (40.4)	68 (38.9)		1
G/A + A/A	90 (59.6)	107 (61.1)	0.865	1.111 (0.705 – 1.750)/ 0.651
**rs368234815 – *IFNL4***	147	171		
**Genotype**				
TT/TT	59 (40.1)	59 (34.5)		1
TT/ΔG	62 (42.2)	75 (45.6)		1.243 (0.749 – 2.062)/ 0.400
ΔG/ΔG	26 (17.7)	34 (19.9)	0.580	1.300 (0.686 – 2.465)/ 0.421

**Allele**				
TT	0.61	0.57		
ΔG	0.39	0.43	0.377	
**Recessive model**				
TT/TT + TT/ΔG	121 (82.3)	137 (80.1)		1
ΔG/ΔG	26 (17.7)	34 (19.9)	0.722	1.157 (0.648 – 2.065)/ 0.621
**Additive model**				
TT/TT	59 (69.4)	59 (63.4)		1
ΔG/ΔG	26 (30.6)	34 (36.6)	0.495	1.315 (0.690 – 2.505)/ 0.405
**Dominant model**				
TT/TT	59 (40.1)	59 (34.5)		1
TT/ΔG + ΔG/ΔG	88 (59.9)	112 (65.5)	0.357	1.260 (0.788 – 2.014)/ 0.334
**rs3775291 – *TLR3***	149	169		
**Genotype**				
C/C	74 (49.7)	77 (45.6)		1

C/T	63 (42.3)	78 (46.1)		1.193 (0.744 – 1.912)/ 0.464
T/T	12 (8.0)	14 (8.3)	0.758	1.214 (0.504 – 2.926)/ 0.665
**Allele**				
C	0.71	0.68		
T	0.29	0.31	0.612	
**Recessive model**				
C/C+ C/T	137 (91.9)	155 (91.7)		1
T/T	12 (8.1)	14 (8.3)	1.000	1.117 (0.477 – 2.615)/ 0.799
**Additive model**				
C/C	74 (86.0)	77 (84.6)		1
T/T	12 (14.0)	14 (15.4)	0.955	1.230 (0.509 – 2.975)/ 0.646
**Dominant model**				
C/C	74 (49.7)	77 (45.6)		1
C/T + T/T	75 (50.3)	92 (54.4)	0.536	1.196 (0.761 – 1.881)/ 0.438
**rs2304256 – *TYK2***	151	174		

**Genotype**				
C/C	87 (57.6)	97 (55.8)		1
C/A	57 (37.8)	66 (37.9)		1.066 (0.665 – 1.709)/ 0.792
A/A	7 (4.6)	11 (6.3)	0.792	1.541 (0.545 – 4.359)/ 0.415
**Allele**				
C	0.76	0.75		
A	0.24	0.25	0.664	
**Dominant model**				
C/C	87 (57.6)	97 (55.7)		1
C/A + A/A	64 (42.4)	77 (44.3)	0.821	1.116 (0.709 – 1.757)/ 0.635
	**Survivors (controls)**	**Non-survivors (cases)**	**Unadjusted P***	**Adjusted OR (95% IC) /† P**
**rs1799752 /*ACE1***	238	69		
**Genotype**				
Del/Del	70 (29.4)	16 (23.2)		1

Del/Ins	164 (68.9)	51 (73.9)		1.717 (0.876 – 3.366)/ 0.115
Ins/Ins	4 (1.7)	2 (2.9)	0.513	1.716 (0.274 – 10.752)/ 0.564
**Allele**				
Del	0.64	0.60		
Ins	0.36	0.40	0.485	
**Dominant model**				
Del/Del	70 (29.4)	16 (23.2)		1
Del/Ins + Ins/Ins	168 (70.6)	53 (76.8)	0.389	1.717 (0.879 – 3.354)/0.114
**rs2285666 – *ACE2***	241	71		
**Genotype**				
C/C	135 (56.0)	42 (59.2)		1
C/T	93 (38.6)	26 (36.6)		0.924 (0.516 – 1.654)/ 0.791
T/T	13 (5.4)	3 (4.2)	0.862	0.745 (0.196 – 2.834)/ 0.666
**Allele**				
C	0.75	0.77		

T	0.25	0.23	0.678	
**Dominant model**				
C/C	135 (56.0)	42 (59.2)		1
C/T + T/T	106 (44.0)	29 (40.8)	0.739	0.901 (0.514 – 1.581)/ 0.717
**rs2109069 – *DPP9***	241	71		
**Genotype**				
G/G	120 (49.8)	35 (49.3)		1
G/A	101 (41.9)	33 (46.5)		1.073 (0.604 – 1.905)/ 0.810
A/A	20 (8.3)	3 (4.2)	0.471	0.626 (0.170 – 2.305)/ 0.481
**Allele**				
G	0.71	0.73		
A	0.29	0.27	0.758	
**Dominant model**				
G/G	120 (49.8)	35 (49.3)		1
G/A + A/A	121 (50.2)	36 (50.7)	1.000	1.009 (0.577 – 1.765)/ 0.975

**rs223675 – *IFNAR2***	241	71		
**Genotype**				
**G/G**	103 (42.8)	24 (33.8)		1
G/A	102 (42.3)	39 (54.9)		1.775 (0.967 – 3.257)/ 0.064
A/A	36 (14.9)	8 (11.3)	0.171	0.988 (0.394 – 2.481)/ 0.980
**Allele**				
**G**	0.64	0.61		
A	0.36	0.39	0.636	
**Recessive model**				
G/G + G/A	205 (85.1)	63 (88.7)		1
A/A	36 (14.9)	8 (11.3)	0.557	0.721 (0.309 – 1.685)/ 0.450
**Additive model**				
G/G	103 (74.1)	24 (75.0)		1
A/A	36 (25.9)	8 (25.0)	1.000	0.983 (0.382 – 2.528)/ 0.972
**Dominant model**				

G/G	103 (42.7)	24 (33.8)		1
G/A + A/A	138 (57.3)	47 (66.2)	0.226	1.562 (0.875 – 2.789)/ 0.132
**rs368234815 – *IFNL4***	236	69		
**Genotype**				
TT/TT	84 (35.6)	27 (39.1)		1
TT/ΔG	108 (45.8)	28 (40.6)		0.845 (0.450 – 1.586)/ 0.600
ΔG/ΔG	44 (18.6)	14 (20.3)	0.748	0.978 (0.451 – 2.120)/ 0.955
**Allele**				
TT	0.58	0.59		
ΔG	0.42	0.41	0.920	
**Recessive model**				
TT/TT + TT/ΔG	192 (81.4)	55 (79.7)		1
ΔG/ΔG	44 (18.6)	14 (20.3)	0.895	1.071 (0.533 – 2.153)/ 0.847
**Additive model**				
TT/TT	84 (65.6)	27 (65.9)		1

ΔG/ΔG	44 (34.4)	14 (34.1)	1.000	0.977 (0.448 – 2.128)/ 0.953
**Dominant model**				
TT/TT	84 (35.6)	27 (39.1)		1
TT/ΔG + ΔG/ΔG	152 (64.4)	42 (60.9)	0.693	0.885 (0.496 – 1.580)/ 0.679
**rs3775291 – *TLR3***	237	69		
**Genotype**				
C/C	116 (48.9)	30 (43.5)		1
C/T	103 (43.5)	33 (47.8)		1.216 (0.678 – 2.181)/ 0.512
T/T	18 (7.6)	6 (8.7)	0.724	1.074 (0.370 – 3.117)/ 0.895
**Allele**				
C	0.71	0.67		
T	0.29	0.33	0.525	
**Recessive model**				
C/C+ C/T	219 (92.4)	63 (91.3)		1
T/T	18 (7.6)	6 (8.7)	0.964	0.976 (0.350 – 2.717)/ 0.962

**Additive model**				
C/C	116 (86.6)	30 (83.3)		1
T/T	18 (13.4)	6 (16.7)	0.822	1.088 (0.378 – 3.129)/ 0.876
**Dominant model**				
C/C	116 (48.9)	30 (43.5)		1
C/T + T/T	121 (51.1)	39 (56.5)	0.507	1.193 (0.680 – 2.093)/ 0.538

Data are shown as number (%) or proportion. *P-values were calculated using χ^2 tests. † P-values and OR (95% CI) obtained using logistic regression analyses adjusting for age and ethnicity. Additive and recessive models were not analyzed for polymorphisms with low frequency of the mutated allele.

Supplementary Table S3. Genotype and allele frequencies of the analyzed polymorphisms in males with COVID-19 according to severity and mortality.

	Inpatients	ICU patients	Unadjusted P*	Adjusted OR (95% IC) /† P
**rs1799752 /*ACE1***	128	232		
**Genotype**				
Del/Del	30 (23.5)	77 (33.2)	0.069	1
Del/Ins	95 (74.2)	145 (62.5)		0.587 (0.353 – 0.974)/ 0.039
Ins/Ins	3 (2.3)	10 (4.3)		1.123 (0.283 – 4.454)/0.869
**Allele**				
Del	0.61	0.64	0.339	-
Ins	0.39	0.36		
**Dominant model**				
Del/Del	30 (23.4)	77 (33.2)	0.069	1
Del/Ins + Ins/Ins	98 (76.6)	155 (66.8)		0.603 (0.364 – 0.999)/ 0.050
**rs2285666 – *ACE2*‡**	128	231		

**Genotype**				
C/C	103 (80.5)	176 (76.2)	0.423	
C/T	-	-		
T/T	25 (19.5)	55 (23.8)		-
**Allele**				
C	0.80	0.76	0.220	
T	0.20	0.24		
**rs2109069 – *DPP9***	129	236		
**Genotype**				
G/G	65 (50.4)	121 (51.2)	0.260	1
G/A	55 (42.6)	87 (36.9)		0.787 (0.492 – 1.258)/ 0.317
A/A	9 (7.0)	28 (11.9)		1.455 (0.639 – 3.314)/ 0.371
**Allele**				
G	0.72	0.70	0.630	

A	0.28	0.30		
**Recessive model**				
G/G + G/A	120 (93.0)	208 (88.1)	0.194	1
A/A	9 (7.0)	28 (11.9)		1.624 (0.735 – 3.588)/ 0.231
**Additive model**				
G/G	65 (87.8)	121 (81.2)	0.288	1
A/A	9 (12.2)	28 (18.8)		1.506 (0.661 – 3.430)/ 0.329
**Dominant model**				
G/G	65 (50.4)	121 (51.3)	0.959	1
G/A + A/A	64 (49.6)	115 (48.7)		0.883 (0.565 – 1.380)/ 0.585
**rs1990760 – *IFIH1***	127	229		
**Genotype**				
C/C	34 (26.8)	67 (29.3)	0.777	1
C/T	65 (51.2)	118 (51.5)		0.784 (0.456 – 1.347)/ 0.379
T/T	28 (22.0)	44 (19.2)		0.660 (0.341 – 1.277)/ 0.217

**Allele**				
C	0.52	0.55	0.546	
T	0.48	0.45		
**Recessive model**				
C/C+ C/T	99 (78.0)	185 (80.8)	0.617	1
T/T	28 (22.0)	44 (19.2)		0.777 (0.449 – 1.344)/ 0.366
**Additive model**				
C/C	34 (54.8)	67 (60.4)	0.585	1
T/T	28 (45.2)	44 (39.6)		0.756 (0.384 – 1.489)/ 0.419
**Dominant model**				
C/C	34 (26.8)	67 (29.3)	0.707	1
C/T + T/T	93 (73.2)	162 (70.7)		0.747 (0.446 – 1.251)/ 0.268
**rs223675 – *IFNAR2***	128	235		
**Genotype**				
**G/G**	63 (49.2)	106 (45.1)	0.735	1

G/A	52 (40.6)	105 (44.7)		1.073 (0.672 – 1.714)/ 0.769
A/A	13 (10.2)	24 (10.2)		1.050 (0.494 – 2.233)/ 0.899
**Allele**				
**G**	0.70	0.67	0.622	
A	0.30	0.33		
**Recessive model**				
G/G + G/A	115 (89.8)	211 (89.8)	1.000	1
A/A	13 (10.2)	24 (10.2)		1.016 (0.493 – 2.090)/ 0.967
**Additive model**				
**G/G**	63 (82.9)	106 (81.5)	0.955	1
A/A	13 (17.1)	24 (18.5)		1.046 (0.491 – 2.231)/ 0.907
**Dominant model**				
**G/G**	63 (49.2)	106 (45.1)	0.522	1
G/A + A/A	65 (50.8)	129 (54.9)		1.068 (0.686 – 1.664)/ 0.770
**rs368234815 – *IFNL4***	127	233		

**Genotype**				
**TT/TT**	51 (40.2)	92 (39.5)	0.854	1
TT/ΔG	55 (43.3)	97 (41.6)		0.960 (0.589 – 1.564)/ 0.869
ΔG/ΔG	21 (16.5)	44 (18.9)		1.215 (0.640 – 2.304)/ 0.552
**Allele**				
**TT**	0.62	0.60	0.751	
ΔG	0.38	0.40		
**Recessive model**				
TT/TT + TT/G	106 (83.5)	189 (81.1)	0.682	1
ΔG/ΔG	21 (16.5)	44 (18.9)		1.240 (0.688 – 2.237)/ 0.474
**Additive model**				
**TT/TT**	51 (70.8)	92 (67.6)	0.753	1
ΔG/ΔG	21 (29.2)	44 (32.4)		1.243 (0.650 – 2.377)/ 0.510
**Dominant model**				
**TT/TT**	51 (40.2)	92 (39.5)	0.991	1

TT/ΔG + ΔG/ΔG	76 (59.8)	141 (60.5)		1.029 (0.654 – 1.617)/ 0.902
**rs3775291 – *TLR3***	129	235		
**Genotype**				
C/C	62 (48.0)	106 (45.1)	0.626	1
C/A	49 (38.0)	101 (43.0)		1.127 (0.700 – 1.814)/ 0.623
A/A	18 (14.0)	28 (11.9)		0.748 (0.374 – 1.495)/ 0.411
**Allele**				
C	0.67	0.67	0.965	
A	0.33	0.33		
**Recessive model**				
C/C+ C/A	111 (86.0)	207 (88.1)	0.693	1
A/A	18 (14.0)	28 (11.9)		0.706 (0.367 – 1.359)/ 0.298
**Additive model**				
C/C	62 (77.5)	106 (79.1)	0.917	1
A/A	18 (22.5)	28 (20.9)		0.772 (0.386 – 1.547)/ 0.466

**Dominant model**				
C/C	62 (48.1)	106 (45.1)	0.666	1
C/A + A/A	67 (51.9)	129 (54.9)		1.024 (0.657 – 1.596)/ 0.916
**rs12329760 – *TMPRSS2***	129	235		
**Genotype**				
C/C	83 (64.3)	159 (67.7)	0.355	1
C/T	39 (30.3)	70 (29.8)		0.885 (0.547 – 1.435)/ 0.621
T/T	7 (5.4)	6 (2.5)		0.473 (0.151 – 1.480)/ 0.198
**Allele**				
C	0.79	0.83	0.353	
T	0.21	0.17		
**Dominant model**				
C/C	83 (64.3)	159 (67.7)	0.599	1
C/T + T/T	46 (35.7)	76 (32.3)		0.824 (0.520 - 1.308)/ 0.412
**rs2304256 – *TYK2***	128	234		

**Genotype**				
C/C	74 (57.8)	128 (54.7)		1
C/T	50 (39.1)	92 (39.3)	0.471	0.999 (0.632 – 1.579)/ 0.996
T/T	4 (3.1)	14 (6.0)		1.754 (0.549 – 5.608)/ 0.343
**Allele**				
C	0.77	0.74	0.423	
T	0.23	0.26		
**Dominant model**				
C/C	74 (57.8)	128 (54.7)	0.646	1
C/T + T/T	54 (42.2)	106 (45.3)		1.056 (0.677 – 1.649)/ 0.809

	**Survivors (controls)**	**Non-survivors (cases)**	**Unadjusted P***	**Adjusted OR (95% IC) /† P**
**rs1799752 /*ACE1***	225	107		
**Genotype**				
Del/Del	62 (27.5)	33 (30.8)	0.298	1

Del/Ins	157 (69.8)	68 (63.6)		0.689 (0.400 – 1.188)/ 0.181
Ins/Ins	6 (2.7)	6 (5.6)		1.249 (0.326 – 4.789)/ 0.746
**Allele**				
Del	0.62	0.63	0.965	
Ins	0.38	0.37		
**Dominant model**				
Del/Del	62 (27.6)	33 (30.8)	0.625	1
Del/Ins + Ins/Ins	163 (72.4)	74 (69.2)		0.710 (0.413 – 1.219)/ 0.214
**rs2285666 – *ACE2*‡**	222	109		
**Genotype**				
C/C	173 (77.9)	89 (81.7)	0.522	
C/T	-	-		
T/T	49 (22.1)	20 (18.3)		
**Allele**				
C	0.78	0.82	0.316	

T	0.22	0.18		
**rs2109069 – *DPP9***	226	111		
**Genotype**				
G/G	121 (53.5)	52 (46.8)	0.154	1
G/A	90 (39.8)	45 (40.6)		1.162 (0.693 – 1.943)/ 0.570
A/A	15 (6.7)	14 (12.6)		1.852 (0.789 – 4.347)/ 0.157
**Allele**				
G	0.73	0.67	0.105	
A	0.27	0.33		
**Recessive model**				
G/G + G/A	211 (93.4)	97 (87.4)	0.103	1
A/A	15 (6.6)	14 (12.6)		1.728 (0.763 – 3.916)/ 0.190
**Additive model**				
G/G	121 (89.0)	52 (78.8)	0.085	1
A/A	15 (11.0)	14 (21.2)		1.924 (0.827 – 4.475)/ 0.129

**Dominant model**				
G/G	121 (53.5)	52 (46.8)	0.299	1
G/A + A/A	105 (46.5)	59 (53.2)		1.268 (0.778 – 2.068)/ 0.341
**rs1990760 – *IFIH1***	223	105		
**Genotype**				
C/C	63 (28.3)	36 (34.3)	0.535	1
C/T	115 (51.6)	49 (46.7)		0.683 (0.387 – 1.206)/ 0.189
T/T	45 (20.1)	20 (19.0)		0.784 (0.380 – 1.618)/ 0.510
**Allele**				
C	0.54	0.58	0.437	
T	0.46	0.42		
**Recessive model**				
C/C+ C/T	178 (79.8)	85 (81.0)	0.927	1
T/T	45 (20.2)	20 (19.0)		0.996 (0.529 – 1.874)/ 0.990
**Additive model**				

C/C	63 (58.3)	36 (64.3)	0.568	1
T/T	45 (41.7)	20 (35.7)		0.832 (0.399 – 1.733)/ 0.623
**Dominant model**				
C/C	63 (28.3)	36 (34.3)	0.326	1
C/T + T/T	160 (71.7)	69 (65.7)		0.709 (0.414 – 1.214)/ 0.210
**rs223675 – *IFNAR2***	225	110		
**Genotype**				
**G/G**	109 (48.4)	49 (44.5)	0.776	1
G/A	93 (41.3)	48 (43.6)		0.996 (0.593 – 1.673)/ 0.989
A/A	23 (10.3)	13 (11.9)		1.140 (0.515 – 2.524)/ 0.747
**Allele**				
**G**	0.69	0.66	0.529	
A	0.31	0.34		
**Recessive model**				
G/G + G/A	202 (89.8)	97 (88.2)	0.799	1

A/A	23 (10.2)	13 (11.8)		1.142 (0.536 – 2.431)/ 0.731
**Additive model**				
**G/G**	109 (82.6)	49 (79.0)	0.694	1
A/A	23 (17.4)	13 (21.0)		1.139 (0.517 – 2.509)/ 0.746
**Dominant model**				
**G/G**	109 (48.4)	49 (44.5)	0.579	1
G/A + A/A	116 (51.6)	61 (55.5)		1.026 (0.630 – 1.670)/ 0.919
**rs368234815 – *IFNL4***	224	108		
**Genotype**				
TT/TT	86 (38.4)	45 (41.7)	0.388	1
TT/ΔG	93 (41.5)	48 (44.4)		0.990 (0.581 – 1.687)/ 0.972
ΔG/ΔG	45 (20.1)	15 (13.9)		0.674 (0.324 – 1.402)/ 0.291
**Allele**				
TT	0.59	0.64	0.278	
ΔG	0.41	0.36		

**Recessive model**				
TT/TT + TT/ΔG	179 (79.9)	93 (86.1)	0.221	1
ΔG/ΔG	45 (20.1)	15 (13.9)		0.678 (0.344 – 1.336)/ 0.261
**Additive model**				
TT/TT	86 (65.6)	45 (75.0)	0.261	1
ΔG/ΔG	45 (34.4)	15 (25.0)		0.663 (0.318 – 1.383)/ 0.273
**Dominant model**				
TT/TT	86 (38.4)	45 (41.7)	0.651	1
TT/ΔG + ΔG/ΔG	138 (61.6)	63 (58.3)		0.891 (0.542 – 1.466)/ 0.650
**rs3775291 – *TLR3***	226	110		
**Genotype**				
C/C	108 (47.8)	49 (44.6)	0.827	1
C/T	91 (40.3)	46 (41.8)		1.125 (0.668 – 1.896)/ 0.658
T/T	27 (11.9)	15 (13.6)		1.127 (0.520 – 2.446)/ 0.762
**Allele**				

C	0.68	0.65	0.581	
T	0.32	0.35		
**Recessive model**				
C/C+ C/T	199 (88.1)	95 (86.4)	0.792	1
T/T	27 (11.9)	15 (13.6)		1.064 (0.513 – 2.207)/ 0.869
**Additive model**				
C/C	108 (80.0)	49 (76.6)	0.712	1
T/T	27 (20.0)	15 (23.4)		1.070 (0.497 – 2.306)/ 0.862
**Dominant model**				
C/C	108 (47.8)	49 (44.5)	0.658	1
C/T + T/T	118 (52.2)	61 (55.5)		1.126 (0.689 – 1.839)/ 0.637
**rs12329760 – *TMPRSS2***	225	111		
**Genotype**				
C/C	148 (65.8)	73 (65.8)	0.720	1
C/T	67 (29.8)	35 (31.5)		0.951 (0.563 – 1.605)/ 0.851

T/T	10 (4.4)	3 (2.7)		0.692 (0.174 – 2.747)/ 0.601
**Allele**				
C	0.81	0.82	0.869	
T	0.19	0.18		
**Dominant model**				
C/C	148 (65.8)	73 (65.8)	1.000	1
C/T + T/T	77 (34.2)	38 (34.2)		0.922 (0.556 – 1.530)/ 0.753
**rs2304256 – *TYK2***	223	111		
**Genotype**				
C/C	125 (56.1)	60 (54.1)	0.835	1
C/A	89 (39.9)	45 (40.5)		1.081 (0.654 – 1.788)/ 0.761
A/A	9 (4.0)	6 (5.4)		1.181 (0.372 – 3.754)/ 0.778
**Allele**				
C	0.76	0.74	0.703	
A	0.24	0.26		

**Dominant model**				
C/C	125 (56.1)	60 (54.1)	0.818	1
C/A + A/A	98 (43.9)	51 (45.9)		1.091 (0.671 – 1.776)/ 0.725

Data are shown as number (%) or proportion. *P-values were calculated using χ^2 tests. P-values and OR (95% CI) obtained using logistic regression analyses adjusting for age and ethnicity. ‡*ACE2* gene is located on the X chromosome, thus men carry only one copy of this gene. Therefore, we did not analyzed genetic models for this polymorphism in men. Additive and recessive models were not analyzed for polymorphisms with low frequency of the mutated allele.

Supplementary Table S4. Genotype and allele frequencies of polymorphisms in non-white patients with COVID-19 classified according to disease severity and mortality.

	**Inpatients**	**ICU patients**	**Unadjusted P***	**Adjusted OR (95% IC) /† P**
**rs1799752 /*ACE1***	84	86		
**Genotype**				
Del/Del	24 (28.6)	25 (29.1)		‡
Del/Ins	60 (71.4)	58 (67.4)		
Ins/Ins	0 (0.0)	3 (3.5)	0.220	
**Allele**				
Del	0.64	0.63		
Ins	0.36	0.37	0.862	
**Dominant model**				
Del/Del	24 (28.6)	25 (29.1)		1
Del/Ins + Ins/Ins	60 (71.4)	61 (70.9)	1.000	0.970 (0.499 -1.887)/ 0.930
**rs2285666 – *ACE2***	84	87		

**Genotype**				
C/C	59 (70.2)	62 (71.3)		1
C/T	20 (23.8)	15 (17.2)		0.636 (0.268 – 1.506)/ 0.303
T/T	5 (6.0)	10 (11.5)	0.301	2.021 (0.638 – 6.408)/ 0.232
**Allele**				
C	0.82	0.80		
T	0.18	0.20	0.693	
**Dominant model**				
C/C	59 (70.2)	62 (71.3)		1
C/T + T/T	25 (29.8)	25 (28.7)	1.000	0.971 (0.488 – 1.933)/ 0.933
**rs2109069 – *DPP9***	85	89		
**Genotype**				
G/G	53 (62.4)	54 (60.6)		1
G/A	28 (32.9)	28 (31.5)		0.990)0.517 – 1.893)/ 0.975

A/A	4 (4.7)	7 (7.9)	0.692	1.730 (0.478 – 6.265)/ 0.404
**Allele**				
G	0.79	0.76		
A	0.21	0.24	0.680	
**Dominant model**				
G/G	53 (62.4)	54 (60.7)		1
G/A + A/A	32 (37.6)	35 (39.3)	0.943	1.082 (0.586 – 1.998)/ 0.800
**rs1990760 – *IFIH1***	82	86		
**Genotype**				
C/C	42 (51.2)	46 (53.5)		1
C/T	31 (37.8)	28 (32.5)		0.814 (0.418 – 1.586)/ 0.546
T/T	9 (11.0)	12 (14.0)	0.716	1.217 (0.465 – 3.183)/ 0.690
**Allele**				
C	0.70	0.70		
T	0.30	0.30	0.961	

**Recessive model**				
C/C+ C/T	73 (89.0)	74 (86.0)		1
T/T	9 (11.0)	12 (14.0)	0.726	1.319 (0.523 – 3.327)/ 0.557
**Additive model**				
C/C	42 (82.4)	46 (79.3)		1
T/T	9 (17.6)	12 (20.7)	0.874	1.232 (0.466 – 3.257)/ 0.674
**Dominant model**				
C/C	42 (51.2)	46 (53.5)		1
C/T + T/T	40 (48.8)	40 (46.5)	0.889	0.906 (0.493 – 1.667)/ 0.751
**rs368234815 – *IFNL4***	84	87		
**Genotype**				
TT/TT	38 (45.2)	28 (32.2)	0.214	1
TT/ΔG	27 (32.2)	34 (39.1)		1.717 (0.849 – 3.472)/ 0.132
ΔG/ΔG	19 (22.6)	25 (28.7)		1.790 (0.827 – 3.872)/ 0.139
**Allele**				

TT	0.61	0.52	0.075	-
ΔG	0.39	0.48		
**Recessive model**				
TT/TT + TT/ΔG	65 (77.4)	62 (71.3)	0.459	1
ΔG/ΔG	19 (22.6)	25 (28.7)		1.379 (0.691 – 2.751)/ 0.362
**Additive model**				
TT/TT	38 (66.7)	28 (52.8)	0.199	1
ΔG/ΔG	19 (33.3)	25 (47.2)		1.783 (0.824 – 3.861)/ 0.142
**Dominant model**				
TT/TT	38 (45.2)	28 (32.2)	0.111	1
TT/ΔG + ΔG/ΔG	46 (54.8)	59 (67.8)		1.747 (0.937 – 3.259)/ 0.079
**rs3775291 – *TLR3***	84	88		
**Genotype**				
C/C	45 (53.5)	48 (54.6)		1
C/T	36 (42.9)	36 (40.9)		0.946 (0.510 – 1.757)/ 0.861

T/T	3 (3.6)	4 (4.5)	0.929	1.254 (0.265 – 5.931)/ 0.776
**Allele**				
C	0.75	0.75		
T	0.25	0.25	0.900	
**Dominant model**				
C/C	45 (53.6)	48 (54.5)		1
C/T + T/T	39 (46.4)	40 (45.5)	1.000	0.970 (0.531 – 1.773)/ 0.922
**rs12329760 – *TMPRSS2***	85	89		
**Genotype**				
C/C	60 (70.6)	54 (60.7)		1
C/T	21 (24.7)	32 (36.0)		1.708 (0.880 – 3.318)/ 0.114
T/T	4 (4.7)	3 (3.3)	0.266	0.827 (0.177 – 3.868)/ 0.809
**Allele**				
C	0.83	0.79		
T	0.17	0.21	0.379	

**Dominant model**				
C/C	60 (70.6)	54 (60.7)		1
C/T + T/T	25 (29.4)	35 (39.3)	0.224	1.565 (0.832 – 2.947)/ 0.165
**rs2304256 – *TYK2***	85	89		
**Genotype**				
C/C	49 (57.6)	55 (61.8)		1
C/A	34 (40.0)	30 (33.7)		0.781 (0.416 – 1.467)/ 0.442
A/A	2 (2.4)	4 (4.5)	0.557	1.802 (0.314 – 10.341)/ 0.509
**Allele**				
C	0.78	0.79		
A	0.22	0.21	0.922	
**Dominant model**				
C/C	49 (57.6)	55 (61.8)		1
C/A + A/A	36 (42.4)	34 (38.2)	0.687	0.837 (0.454 – 1.545)/ 0.569
	**Survivors (controls)**	**Non-survivors**	**Unadjusted P***	**Adjusted OR (95% IC) /† P**

		(cases)		
**rs2285666 – *ACE2***	123	42		
**Genotype**				
C/C	88 (71.5)	30 (71.4)		1
C/T	27 (22.0)	7 (16.7)		1.021 (0.334 – 3.121)/ 0.972
T/T	8 (6.5)	5 (11.9)	0.453	1.301 (0.365 – 4.631)/ 0.685
**Allele**				
C	0.83	0.80		
T	0.17	0.20	0.687	
**Dominant model**				
C/C	88 (71.5)	30 (71.4)		1
C/T + T/T	35 (28.5)	12 (28.6)	1.000	1.132 (0.480 – 2.673)/ 0.777
**rs2109069 – *DPP9***	124	44		
**Genotype**				
G/G	77 (62.0)	27 (61.4)		1

G/A	39 (31.5)	15 (34.1)		1.133 (0.523 – 2.458)/ 0.751
A/A	8 (6.5)	2 (4.5)	0.873	0.810 (0.149 – 4.398)/ 0.810
**Allele**				
G	0.78	0.78		
A	0.22	0.22	0.971	
**Dominant model**				
G/G	77 (62.1)	27 (61.4)		1
G/A + A/A	47 (37.9)	17 (38.6)	1.000	1.082 (0.516 – 2.270)/ 0.834
**rs1990760 – *IFIH1***	121	42		
**Genotype**				
C/C	59 (48.8)	26 (61.9)	0.313	1
C/T	46 (38.0)	11 (26.2)		0.389 (0.162 – 0.931)/ 0.034
T/T	16 (13.2)	5 (11.9)		0.714 (0.224 – 2.270)/ 0.568
**Allele**				
C	0.68	0.75	0.240	

T	0.32	0.25		
**Recessive model**				
C/C+ C/T	105 (86.8)	37 (88.1)	1.000	1
T/T	16 (13.2)	5 (11.9)		0.999 (0.328 – 3.042)/ 0.999
**Additive model**				
C/C	59 (78.7)	26 (83.9)	0.731	1
T/T	16 (21.3)	5 (16.1)		0.729 (0.220 – 2.412)/ 0.604
**Dominant model**				
C/C	59 (48.8)	26 (61.9)	0.197	1
C/T + T/T	62 (51.2)	16 (38.1)		0.464 (0.215 – 1.002)/ 0.051
**rs368234815 – *IFNL4***	122	43		
**Genotype**				
**TT/TT**	49 (40.1)	14 (32.6)		1
TT/ΔG	45 (36.9)	15 (34.8)		1.114 (0.469 – 2.647)/ 0.807
ΔG/ΔG	28 (23.0)	14 (32.6)	0.434	1.660 (0.665 – 4.139)/ 0.277

**Allele**				
TT	0.59	0.50		
ΔG	0.41	0.50	0.209	
**Recessive model**				
TT/TT + TT/ΔG	94 (77.0)	29 (67.4)		1
ΔG/ΔG	28 (23.0)	14 (32.6)	0.298	1.572 (0.706 – 3.499)/ 0.268
**Additive model**				
TT/TT	49 (63.6)	14 (50.0)		1
ΔG/ΔG	28 (36.4)	14 (50.0)	0.300	1.647 (0.650 – 4.175)/ 0.293
**Dominant model**				
TT/TT	49 (40.2)	14 (32.6)		1
TT/ΔG + ΔG/ΔG	73 (59.8)	29 (67.4)	0.484	1.322 (0.616 – 2.838)/ 0.473
**rs3775291 – *TLR3***	123	44		
**Genotype**				
C/C	69 (56.1)	22 (50.0)		1

C/T	48 (39.0)	21 (47.7)		1.564 (0.742 – 3.295)/ 0.240
T/T	6 (4.9)	1 (2.3)	0.512	0.612 (0.066 – 5.637)/ 0.665
**Allele**				
C	0.76	0.74		
T	0.24	0.26	0.855	
**Dominant model**				
C/C	69 (56.1)	22 (50.0)		1
C/T + T/T	54 (43.9)	22 (50.0)	0.603	1.456 (0.701 – 3.021)/ 0.314
**rs2304256 – *TYK2***	124	44		
**Genotype**				
C/C	73 (58.9)	27 (61.4)		1
C/A	46 (37.1)	16 (36.4)		1.048 (0.491 – 2.234)/ 0.904
A/A	5 (4.0)	1 (2.2)	0.852	0.717 (0.076 – 6.799)/ 0.772
**Allele**				
C	0.77	0.80		

A	0.23	0.20	0.792	
**Dominant model**				
C/C	73 (58.9)	27 (61.4)		1
C/A + A/A	51 (41.1)	17 (38.6)	0.912	1.018 (0.486 – 2.133)/ 0.963

Data are shown as number (%) or proportion. *P-values were calculated using χ^2 tests. † P-values and OR (95% CI) obtained using logistic regression analyses adjusting for age and sex. ‡Logistic regression cannot be performed because the frequency of the Ins/Ins genotype is zero in the inpatients group. Additive and recessive models were not analyzed for polymorphisms with low frequency of the mutated allele.

Supplementary Table S5. Genotype and allele frequencies of the nine polymorphisms of interest in white patients with COVID-19.

	Inpatients	ICU patients	Unadjusted P*	Adjusted OR (95% IC) /† P
**rs1799752 /*ACE1***	180	311		
**Genotype**				
Del/Del	57 (31.7)	86 (27.7)	0.374	1
Del/Ins	119 (66.1)	212 (68.1)		1.227 (0.816 – 1.844)/ 0.325
Ins/Ins	4 (2.2)	13 (4.2)		1.983 (0.610 – 6.450)/ 0.255
**Allele**				
Del	0.65	0.62	0.387	
Ins	0.35	0.38		
**Dominant model**				
Del/Del	57 (31.7)	86 (27.7)	0.401	1
Del/Ins + Ins/Ins	123 (68.3)	225 (72.3)		1.254 (0.836 – 1.879)/ 0.274
**rs2285666 – *ACE2***	182	313		
**Genotype**				

C/C	117 (64.3)	215 (68.7)	0.109	1
C/T	38 (20.9)	43 (13.7)		0.758 (0.435 – 1.319)/ 0.326
T/T	27 (14.8)	55 (17.6)		1.048 (0.623 – 1.764)/ 0.859
**Allele**				
C	0.75	0.75	0.828	
T	0.25	0.25		
**Recessive model**				
C/C + C/T	155 (85.2)	258 (82.4)	0.506	1
T/T	27 (14.8)	55 (17.6)		1.079 (0.643 – 1.810)/ 0.775
**Additive model**				
C/C	117 (81.3)	215 (79.6)	0.791	1
T/T	27 (18.8)	55 (20.4)		1.053 (0.625 – 1.775)/ 0.845
**Dominant model**				
C/C	117 (64.3)	215 (68.7)	0.365	1
C/T + T/T	65 (35.7)	98 (31.3)		0.903 (0.607 – 1.343)/ 0.613

**rs2109069 – *DPP9***	182	316		
**Genotype**				
G/G	80 (44.0)	144 (45.6)	0.592	1
G/A	86 (47.2)	137 (43.4)		0.906 (0.615 – 1.335)/ 0.619
A/A	16 (8.8)	35 (11.0)		1.198 (0.620 – 2.313)/ 0.591
**Allele**				
G	0.67	0.67	0.969	
A	0.33	0.33		
**Recessive model**				
G/G + G/A	166 (91.2)	281 (88.9)	0.512	1
A/A	16 (8.8)	35 (11.1)		1.258 (0.671 – 2.358)/ 0.474
**Additive model**				
G/G	80 (83.3)	144 (80.4)	0.671	1
A/A	16 (16.7)	35 (19.6)		1.173 (0.607 – 2.267)/ 0.635

**Dominant model**				
G/G	80 (44.0)	144 (45.6)	0.799	1
G/A + A/A	102 (56.0)	172 (54.4)		0.953 (0.658 – 1.380)/ 0.798
**rs1990760 – *IFIH1***	179	309		
**Genotype**				
C/C	49 (27.4)	74 (23.9)	0.687	1
C/T	91 (50.8)	162 (52.4)		1.146 (0.732 – 1.794)/ 0.551
T/T	39 (21.8)	73 (23.7)		1.246 (0.729 – 2.130)/ 0.421
**Allele**				
C	0.53	0.50	0.467	
T	0.47	0.50		
**Recessive model**				
C/C+ C/T	140 (78.2)	236 (76.4)	0.724	1
T/T	39 (21.8)	73 (23.6)		1.138 (0.729 – 1.776)/ 0.570
**Additive model**				

C/C	49 (55.7)	74 (50.3)	0.510	1
T/T	39 (44.3)	73 (49.7)		1.260 (0.737 – 2.157)/ 0.399
**Dominant model**				
C/C	49 (27.4)	74 (23.9)	0.464	1
C/T + T/T	130 (72.6)	235 (76.1)		1.176 (0.769 – 1.798)/ 0.455
**rs223675 – *IFNAR2***	182	315		
**Genotype**				
**G/G**	75 (41.2)	131 (41.6)	0.392	1
G/A	80 (44.0)	150 (47.6)		1.082 (0.728 – 1.608)/ 0.696
A/A	27 (14.8)	34 (10.8)		0.758 (0.422 – 1.362)/ 0.354
**Allele**				
**G**	0.63	0.65	0.526	
A	0.37	0.35		
**Recessive model**				
G/G + G/A	155 (85.2)	281 (89.2)	0.238	1

A/A	27 (14.8)	34 (10.8)		0.727 (0.420 – 1.257)/ 0.254
**Additive model**				
**G/G**	75 (73.5)	131 (79.4)	0.338	1
A/A	27 (26.5)	34 (20.6)		0.764 (0.423 – 1.380)/ 0.373
**Dominant model**				
**G/G**	75 (41.2)	131 (41.6)	1.000	1
G/A + A/A	107 (58.8)	184 (58.4)		1.002 (0.689 – 1.457)/ 0.992
**rs368234815 – *IFNL4***	177	311		
**Genotype**				
TT/TT	66 (37.3)	120 (38.6)	0.866	1
TT/ΔG	84 (47.5)	140 (45.0)		0.936 (0.622 – 1.407)/ 0.749
ΔG/ΔG	27 (15.2)	51 (16.4)		1.064 (0.608 – 1.861)/ 0.828
**Allele**				
TT	0.61	0.61	0.964	
ΔG	0.39	0.39		

**Recessive model**				
TT/TT + TT/ΔG	150 (84.7)	260 (83.6)	0.839	1
ΔG/ΔG	27 (15.3)	51 (16.4)		1.104 (0.661 – 1.841)/ 0.706
**Additive model**				
**TT/TT**	66 (71.0)	120 (70.2)	1.000	1
ΔG/ΔG	27 (29.0)	51 (29.8)		1.071 (0.610 – 1.880)/ 0.812
**Dominant model**				
**TT/TT**	66 (37.3)	120 (38.6)	0.852	1
TT/ΔG + ΔG/ΔG	111 (62.7)	191 (61.4)		0.967 (0.658 – 1.420)/ 0.864
**rs3775291 – *TLR3***	181	310		
**Genotype**				
C/C	86 (47.5)	134 (43.3)	0.405	1
C/T	70 (38.7)	139 (44.8)		1.253 (0.841 – 1.866)/ 0.268
T/T	25 (13.8)	37 (11.9)		0.873 (0.487 – 1.564)/ 0.647
**Allele**				

C	0.67	0.66	0.752	
T	0.33	0.34		
**Recessive model**				
C/C+ C/T	156 (86.2)	273 (88.1)	0.643	1
T/T	25 (13.8)	37 (11.9)		0.783 (0.451 – 1.359)/ 0.385
**Additive model**				
C/C	86 (77.5)	134 (78.4)	0.978	1
T/T	25 (22.5)	37 (21.6)		0.881 (0.491 – 1.579)/ 0.670
**Dominant model**				
C/C	86 (47.5)	134 (43.2)	0.408	1
C/T + T/T	95 (52.5)	176 (56.8)		1.152 (0.794 – 1.671)/ 0.457
**rs12329760 – *TMPRSS2***	182	315		
**Genotype**				
C/C	121 (66.5)	205 (65.1)	0.890	1
C/T	55 (30.2)	101 (32.0)		1.100 (0.736 – 1.643)/ 0.643

T/T	6 (3.3)	9 (2.9)		0.857 (0.296 – 2.481)/ 0.775
**Allele**				
C	0.82	0.81	0.917	
T	0.18	0.19		
**Dominant model**				
C/C	121 (66.5)	205 (65.1)	0.826	1
C/T + T/T	61 (33.5)	110 (34.9)		1.075 (0.729 – 1.586)/ 0.715
**rs2304256 – *TYK2***	181	313		
**Genotype**				
C/C	105 (58.0)	168 (53.7)	0.463	1
C/A	68 (37.6)	124 (39.6)		1.144 (0.777 – 1.685)/ 0.495
A/A	8 (4.4)	21 (6.7)		1.590 (0.675 – 3.748)/ 0.289
**Allele**				
C	0.77	0.73	0.282	
A	0.23	0.27		

**Recessive model**				
C/C+ C/A	173 (95.6)	292 (93.3)	0.398	1
A/A	8 (4.4)	21 (6.7)		1.506 (0.648 – 3.500)/ 0.341
**Additive model**				
C/C	105 (92.9)	168 (88.9)	0.343	1
A/A	8 (7.1)	21 (11.1)		1.556 (0.658 – 3.680)/ 0.314
**Dominant model**				
C/C	105 (58.0)	168 (53.7)	0.401	1
C/A + A/A	76 (42.0)	145 (46.3)		1.192 (0.821 – 1.731)/ 0.355

	Survivors (controls)	Non-survivors (cases)	Unadjusted P*	Adjusted OR (95% IC) /† P
**rs1799752 /*ACE1***	323	132		
**Genotype**				
Del/Del	89 (27.6)	37 (28.0)	0.922	1
Del/Ins	224 (69.3)	90 (68.2)		1.050 (0.651 – 1.694)/ 0.842

Ins/Ins	10 (3.1)	5 (3.8)		0.841 (0.248 – 2.857)/ 0.782
**Allele**				
Del	0.61	0.62	0.964	
Ins	0.37	0.38		
**Recessive model**				
Del/Del + Del/Ins	313 (96.9)	127 (96.2)	0.932	1
Ins/Ins	10 (3.1)	5 (3.8)		0.813 (0.251 – 2.636)/ 0.731
**Additive model**				
Del/Del	89 (89.9)	37 (88.1)	0.985	1
Ins/Ins	10 (10.1)	5 (11.9)		0.877 (0.259 – 2.967)/ 0.832
**Dominant model**				
Del/Del	89 (27.6)	37 (28.0)	1.000	1
Del/Ins + Ins/Ins	234 (72.4)	95 (72.0)		1.038 (0.646 – 1.669)/ 0.877
**rs2285666 – *ACE2***	323	136		
**Genotype**				

C/C	211 (65.3)	99 (72.8)	0.289	1
C/T	60 (18.6)	19 (14.0)		0.883 (0.44 – 1.753)/ 0.721
T/T	52 (16.1)	18 (13.2)		0.681 (0.396 – 1.256)/ 0.218
**Allele**				
C	0.75	0.80	0.111	
T	0.25	0.20		
**Recessive model**				
C/C + C/T	271 (83.9)	118 (86.8)	0.524	1
T/T	52 (16.1)	18 (13.2)		0.687 (0.373 – 1.265)/ 0.228
**Additive model**				
C/C	211 (80.2)	99 (84.6)	0.382	1
T/T	52 (19.8)	18 (15.4)		0.682 (0.369 – 1.263)/ 0.224
**Dominant model**				
C/C	211 (65.3)	99 (72.8)	0.147	1
C/T + T/T	112 (34.7)	37 (27.2)		0.761 (0.475 – 1.221)/ 0.258

**rs2109069 – *DPP9***	326	136		
**Genotype**				
G/G	151 (46.3)	59 (43.4)	0.613	1
G/A	148 (45.4)	62 (45.6)		1.124 (0.722 – 1.752)/ 0.605
A/A	27 (8.3)	15 (11.0)		1.481 (0.703 – 3.118)/ 0.301
**Allele**				
G	0.69	0.66	0.442	
A	0.31	0.34		
**Recessive model**				
G/G + G/A	299 (91.7)	121 (89.0)	0.448	1
A/A	27 (8.3)	15 (11.0)		1.397 (0.687 – 2.840)/ 0.356
**Additive model**				
G/G	151 (84.4)	59 (79.7)	0.421	1
A/A	27 (15.2)	15 (20.3)		1.484 (0.708 – 3.111)/ 0.296

**Dominant model**				
G/G	151 (46.3)	59 (43.4)	0.635	1
G/A + A/A	175 (53.7)	77 (56.6)		1.179 (0.772 – 1.800)/ 0.447

	**Survivors**	**Non-Survivors**		
**rs1990760 – *IFIH1***	323	130		
**Genotype**				
C/C	84 (26.0)	34 (26.2)	0.412	1
C/T	172 (53.3)	62 (47.6)		0.913 (0.544 – 1.532)/ 0.730
T/T	67 (20.7)	34 (26.2)		1.490 (0.810 – 2.739)/ 0.200
**Allele**				
C	0.53	0.50	0.474	-
T	0.47	0.50		
**Recessive model**				
C/C+ C/T	256 (79.3)	96 (73.8)	0.260	1
T/T	67 (20.7)	34 (26.2)		1.582 (0.955 – 2.621)/ 0.075

**Additive model**				
C/C	84 (55.6)	34 (50.0)	0.531	1
T/T	67 (44.4)	34 (50.0)		1.459 (0.797 – 2.670)/ 0.221
**Dominant model**				
C/C	84 (26.0)	34 (26.2)	1.000	1
C/T + T/T	239 (74.0)	96 (73.8)		1.060 (0.651 – 1.725)/ 0.815
**rs223675 – *IFNAR2***	326	135		
**Genotype**				
**G/G**	136 (41.7)	57 (42.2)	0.281	1
G/A	144 (44.2)	66 (48.9)		1.109 (0.710 – 1.734)/ 0.649
A/A	46 (14.1)	12 (8.9)		0.637 (0.305 – 1.330)/0.230
**Allele**				
**G**	0.64	0.67	0.452	
A	0.36	0.33		
**Recessive model**				

G/G + G/A	280 (85.9)	123 (91.1)	0.166	1
A/A	46 (14.1)	12 (8.9)		0.604 (0.301 – 1.211)/ 0.155
**Additive model**				
**G/G**	136 (74.7)	57 (82.6)	0.248	1
A/A	46 (25.3)	12 (17.4)		0.650 (0.309 – 1.367)/ 0.256
**Dominant model**				
**G/G**	136 (41.7)	57 (42.2)	1.000	1
G/A + A/A	190 (58.3)	78 (57.8)		0.996 (0.650 – 1.526)/ 0.984
**rs368234815 – *IFNL4***	321	132		
**Genotype**				
**TT/TT**	115 (35.8)	56 (42.4)	0.175	1
TT/ΔG	149 (46.4)	61 (46.2)		0.873 (0.551 – 1.382)/ 0.562
ΔG/ΔG	57 (17.8)	15 (11.4)		0.549 (0.279 – 1.082)/ 0.083
**Allele**				
**TT**	0.59	0.65	0.068	

ΔG	0.41	0.35		
**Recessive model**				
TT/TT + TT/ΔG	115 (66.9)	56 (78.9)	0.087	1
ΔG/ΔG	57 (33.1)	15 (21.1)		0.591 (0.315 – 1.112)/ 0.103
**Additive model**				
**TT/TT**	264 (82.2)	117 (88.6)	0.121	1
ΔG/ΔG	57 (17.8)	15 (11.4)		0.543 (0.276 – 1.068)/ 0.077
**Dominant model**				
**TT/TT**	115 (35.8)	56 (42.4)	0.226	1
TT/ΔG + ΔG/ΔG	206 (64.2)	76 (57.6)		0.782 (0.506 – 1.207)/ 0.267
**rs3775291 –** ***TLR3***	323	133		
**Genotype**				
C/C	149 (46.1)	57 (42.9)	0.699	1
C/T	136 (42.1)	57 (42.9)		1.047 (0.664 – 1.650)/ 0.843
T/T	38 (11.8)	19 (14.2)		1.105 (0.571 – 2.139)/ 0.766

**Allele**				
C	0.67	0.64	0.444	
T	0.33	0.36		
**Recessive model**				
C/C+ C/T	285 (88.2)	114 (85.7)	0.559	1
T/T	38 (11.8)	19 (14.3)		1.080 (0.581 – 2.009)/ 0.807
**Additive model**				
C/C	149 (79.7)	57 (75.0)	0.503	1
T/T	38 (20.3)	19 (25.0)		1.128 (0.587 – 2.167)/ 0.718
**Dominant model**				
C/C	149 (46.1)	57 (42.9)	0.593	1
C/T + T/T	174 (53.9)	76 (57.1)		1.061 (0.692 – 1.625)/ 0.787
**rs12329760 – *TMPRSS2***	325	136		
**Genotype**				
C/C	212 (65.2)	91 (66.9)	0.929	1

C/T	102 (31.4)	41 (30.2)		0.973 (0.614 – 1.542)/ 0.908
T/T	11 (3.4)	4 (2.9)		0.834 (0.249 – 2.794)/ 0.768
**Allele**				
C	0.81	0.82	0.776	
T	0.19	0.18		
**Dominant model**				
C/C	212 (65.2)	91 (66.9)	0.811	1
C/T + T/T	113 (34.8)	45 (33.1)		0.959 (0.614 – 1.496)/ 0.853
**rs2304256 – *TYK2***	322	136		
**Genotype**				
C/C	179 (55.6)	74 (54.4)	0.113	1
C/A	130 (40.4)	50 (36.8)		0.983 (0.629 – 1.534)/ 0.939
A/A	13 (4.0)	12 (8.8)		2.026 (0.842 – 4.879)/ 0.115
**Allele**				
C	0.76	0.73	0.385	

A	0.24	0.27		
**Recessive model**				
C/C+ C/A	309 (96.0)	124 (91.2)	0.066	1
A/A	13 (4.0)	12 (8.8)		2.041 (0.864 – 4.822)/ 0.104
**Additive model**				
C/C	179 (93.2)	74 (86.0)	0.088	1
A/A	13 (6.8)	12 (14.0)		2.009 (0.835 – 4.831)/ 0.119
**Dominant model**				
C/C	179 (55.6)	74 (54.4)	0.897	1
C/A + A/A	143 (44.4)	62 (45.6)		1.087 (0.711 – 1.662)/ 0.699

Data are shown as number (%) or proportion. *P-values were calculated using χ^2 tests. † P-values and OR (95% CI) obtained using logistic regression analyses adjusting for age and sex. Additive and recessive models were not analyzed for polymorphisms with low frequency of the mutated allele.
